# Emotion Norms, Display Rules, and Regulation in the Akan Society of Ghana: An Exploration Using Proverbs

**DOI:** 10.3389/fpsyg.2018.01916

**Published:** 2018-10-31

**Authors:** Vivian A. Dzokoto, Annabella Osei-Tutu, Jane J. Kyei, Maxwell Twum-Asante, Dzifa A. Attah, Daniel K. Ahorsu

**Affiliations:** ^1^African American Studies, Virginia Commonwealth University, Richmond, VA, United States; ^2^Department of Psychology, University of Ghana, Legon, Ghana; ^3^Rutgers University, The State University of New Jersey, New Brunswick, NJ, United States; ^4^Department of Psychology, Fayetteville State University, Fayetteville, NC, United States; ^5^Department of Psychiatry, School of Medicine and Dentistry, University of Ghana, Legon, Ghana; ^6^Department of Rehabilitation Sciences, The Hong Kong Polytechnic University, Kowloon, Hong Kong

**Keywords:** culture, emotion, Africa, emotion regulation, emotion display rules

## Abstract

Proverbs are widely used by the Akan of West Africa. The current study thematically analyzed an Akan proverb compendium for proverbs containing emotion references. Of the identified proverbs, a focus on negative emotions was most typical. Emotion-focused proverbs highlighted four emotion regulation strategies: change in cognition, response modulation, situation modification, and situation selection. A subset of proverbs addressed emotion display rules restricting the expression of emotions such as pride, and emotional contagion associated with emotions such as shame. Additional themes including: social context influences on the expression and experience of emotion; expectations of emotion limits; as well as the nature of emotions were present in the proverb collection. In general, Akan emotion-related proverbs stress individual-level responsibility for affect regulation in interpersonal interactions and societal contexts.

## Introduction

Humans experience and express emotion in order to react to, interface with, and adapt to the physical and social environment. Emotion regulation — the ability to manage and modify one’s emotional experiences and expressions (Matsumoto, 2006) — is thus important in everyday life. The adaptation and modification of emotion comprises a heterogeneous set of processes involving changes in the experiential, behavioral, and physiological responses associated with an emotion (Gross and John, 2003). These changes are generally either antecedent-focused or response-focused (Gross, 1998a, 2002), meaning emotions can be modified both before and after their initiation (Liverant et al., 2008). Cultural context has been shown to impact emotion at both of these stages (e.g., Davis et al., 2012; Matsumoto and Juang, 2013; Ma et al., 2017). Because cultural rules are not routinely explicit, it is often difficult to access cultural expectations and preferred practices concerning emotion display and regulation by directly asking participants of the culture what the rules are. This may be particularly true of cultural contexts such as Ghana, where in contrast to many North American settings, the preferred communication style is high context (Hall, 1992); indirectness and symbolism rather than explicit communication is common (Copeland and Griggs, 1986; Yankah, 1995); emotion is considered less important to attend to in everyday life (Dzokoto, 2010); and emotion discourse in the description of emotionally significant positive and negative life events is less elaborate (in terms of total number of emotion words used) (Dzokoto et al., 2013). In such settings, a better approach may be to target the symbolism through which the cultural rules are communicated. The goal of the present study is to identify – through qualitative analysis- cultural rules about emotion regulation in Ghana using a widely used (in Ghana), symbolic cultural artifact that is an important component of everyday discourse, socialization, and intergenerational value transmission (Brookman-Amissah, 1986); the proverb.

### Emotion Regulation: Strategies and Cultural Variation

Gross (1998b) identified several emotion regulation strategies: situation selection, situation modification, attentional deployment, cognition change (antecedent-focused emotion regulation strategies) and response modulation (response-focused emotion regulation strategy). These strategies have been observed in Western as well as non-western cultures. The regulation strategy of situation selection refers to the decision to either approach or avoid certain people, places, or objects in order to regulate emotions. Situation modification is an active effort to change the situation whereas attentional deployment involves devoting efforts to a specific aspect of an emotion situation by distracting, concentrating, or ruminating on it. Cognitive change aims at modifying the emotion response by changing the cognitive evaluation using defenses such as denial, isolation, intellectualization, or use of techniques such as cognitive reframing and reappraisal. According to Gross and John (2003), cognitive reappraisals comprise the interpretation of a potential emotion-eliciting situation in a way that changes its emotional impact. Finally, response modulation entails direct attempts at changing physiological, experiential, or behavioral responses; using techniques like exercise, biofeedback, relaxation, food, and drugs once an emotion has been elicited (Gross, 1998b,c). It may also present as emotional/expressive suppression (Gross and John, 2003), faking, or the amplification of an emotional response (Grandey, 2000; Liverant et al., 2008).

A growing body of research demonstrates that aspects of emotion regulation occur along cultural patterns. Individuals in collectivistic cultures, where social harmony and self-restraint are emphasized, are more likely to regulate their emotion using positive attributions in anger-inducing interpersonal situations (Trommsdorff, 2012). In contrast, individuals in individualistic cultures utilize negative attributions, which activate goals for retaliation and maintenance of self-esteem. Specific cognitive change strategies, for example distancing and thinking about the victim have been found to have a strong association with culture, gender and emotional intensity (Davis et al., 2012). Due to cultural differences in the valuing and meaning of positive and negative emotions, Asians tend not to savor positive emotions, and are less likely to down-regulate negative emotion while European Americans prefer not to dampen positive emotions and are more likely to regulate emotion hedonically in experimental and experiential settings (Miyamoto and Ma, 2011; Miyamoto et al., 2014). Wan and Savina (2015) compared preferred emotion regulation strategies in US and Hong Kong Chinese public school children. They observed that US children endorsed positive distraction and talking to someone to a greater extent than the comparison group, while Chinese (but not US) children endorsed deep breathing as a fear regulation strategy. For both groups, situational avoidance and talking to someone were endorsed for the downregulation of anger. More recently, Ma et al. (2017) observed that culture (Asians from 5 countries and Asian Americans versus EuroAmerican) and situational demands (such as required cognitive effort) interact to shape positive emotion regulation. Nam et al. (2018) noted that whereas for American subjects, the high use of emotion suppression was detrimental to evaluations of life satisfaction, low use of emotion suppression was beneficial to evaluations of life satisfaction for Hong Kong Chinese research participants. In a study of 6 European countries, Potthoff et al. (2016) observed that participants from northern Europe used cognitive emotion regulation strategies as rumination and catastrophization less than respondents from Southern and Eastern Europe. Collectively, work in this area notes cultural and racial differences in mean levels of some emotion regulation strategies, and is beginning to explore association between emotion regulation and psychological adjustment in different cultural settings (Cheung and Park, 2010).

Majority of the studies exploring the interplay of culture and emotion regulation have focused on East–West differences. In contrast, emotion regulation norms and preferences in African settings have been largely understudied. As such, while identified emotion regulation strategies appear to be relevant to markedly different cultural contexts, there is limited information about which ones are promoted and valued in varied African settings. Of the relatively few studies in this area that included African countries, there are some suggestions that African settings may promote unique emotion regulation strategies and patterns. Bozicevic et al. (2016) found cultural differences in emotion regulation between samples of United Kingdom, White South African, and Black South African children, and in maternal responses to infant distress. In contrast, Kliewer et al. (2017) found that similar to research done elsewhere in the world, poverty was significantly associated with emotion dysregulation in South African youth.

### Emotion Display Rules

Some elements of emotion regulation and associated variation across cultures have been explored through the lens of cultural display rules. Emotion display rules (socialized, culturally determined rules concerning the propriety of specific emotion expression at specific times, places, and interpersonal settings) train people to automatically modify (dampen, intensify, neutralize, qualify, mask, and fabricate) elicited emotional reactions according to context-specific demand characteristics (Matsumoto and Juang, 2013). These rules, guided in part by cultural values related to inherent social structures and interpersonal relationships, are important for the preservation of social order; the generation of culturally appropriate behavior and emotional responding; and the facilitation of social functioning (Matsumoto and Juang, 2013).

Recent studies have found some cultural differences in emotion displays and display rules. For instance, Elfenbein et al. (2007) posited the existence of expressive display cultural variation patterns similar to those documented in linguistic dialects. While posed expressions of fear, embarrassment, and disgust were similar in Quebecois and Gabonese research participants, different patterns of muscle activations for the two groups were observed for emotions such as serenity, shame, and contempt. Using the GRID instrument to assess components of emotional meaning, Hess et al. (2013) attribute the aforementioned variation to differences in modal appraisal patterns. Also, Thibault et al. (2012) found that while Quebecois perceive the Duchenne marker as an indicator of an authentic smile, Gabonese do not, and Mainland Chinese immigrants use it only when evaluating smiles of Quebecois.

In work that directly explored social expectations of emotion display, Safdar et al. (2009) found cross-national differences in emotional display rules in a 3-country study (Japan, United States, Canada). In their study, Japanese participants thought they should limit the expression of powerful emotions such as anger, as well as positive emotions such as happiness less than North Americans. Additionally, Japanese participants used interaction partner-dependent display rules when expressing powerful emotions while the other groups did not. Matsumoto et al. (2008) used an adapted version of the Display Rule Assessment Inventory to assess expectations about emotion displays for anger, contempt, fear, disgust, happiness, sadness, and surprise in 32 countries. While they observed large universal effects of emotion display norms especially for negative emotions, they also found that individualism was associated with expression norms for happiness and surprise. Subsequently, from a 33 country study which included 2 African countries (Nigeria and Zimbabwe), Matsumoto et al. (2009) concluded that emotional expression differentiation is an important mechanism through which individuals may learn to behave differently in different contexts. Chung (2012) observed that American research participants were more likely than Japanese participants to endorse expressions of anger, contempt, disgust, fear, happiness, and surprise. However, this difference was partially mediated by United States–Japan differences in self-deceptive enhancement. More recently, Tsai et al. (2016) observed that ideal affect differences (degree of preference for high arousal positive emotion states) between the United States and China were reflected in variations in (i) official photo smiles of top-ranked leaders (politicians, CEOs and university presidents); (ii) smiles of winning and losing political candidates; and (iii) CEOs and university presidents of varying ranks from the two countries. The researchers found similar parallels between ideal affect ratings of college students from 10 Western and Eastern countries and emotional expressions displayed by legislators from each of the 10 countries in their official photographs. Culture-specific emotion communication patterns along several of Hofstede’s cultural dimensions have even been observed in studies of affective communication via social media (Furner and George, 2012; Park et al., 2014). Collectively, the emotion display rule literature indicates the existence of display rule universals, as well as patterns of expectations concerning emotional expression nuanced by culture. Because African countries have only recently began to be featured in this focus of research, there is much to learn about emotion display rules and associated nuances in its diverse geographical settings and ethnolinguistic groups.

### Proverbs as Cultural Product

Psychological experiences can be examined using a variety of modes of enquiry that have been developed, refined, and tested over time. Unfortunately, some of them are not easily deployable or efficient to use in under-researched settings which often have structural and technological access and affordability barriers that hamper mainstream psychology data collection strategies. A potential way of including such populations in psychological discourse, and thus increasing the global footprint of mainstream and cultural psychological inquiry, is harnessing extant and easily accessible assets of such societies that can provide psychological information. A few researchers have demonstrated that cultural products are useful in explaining human behavior within a cultural context (Morling and Lamoreaux, 2008; DeWall et al., 2011). The body of cultural products—which include proverbs, myths, poems, crafts, visual art, songs, folktales, books, magazines, and advertisements—are thought to contain valuable cultural information that symbolizes the uniqueness of a specific society and “carry the footprints of the social context in which they were created” thereby offering “clues to the long-term cultural dynamics in play” (Kesebir and Kesebir, 2017, p. 259). In other words, cultural products represent cultural affordances (Salter and Adams, 2016). Thus, cultural product (artifact) analysis allows for an exploration of the dynamic interplay between community-relevant events and of psychological phenomena and between facts and incidents across different historical periods (Curşeu and Pop-Curşeu, 2011). Cultural artifact analysis can offer an understanding of (i) cross cultural, within-cultural, and within-person differences (DeWall et al., 2011) and (ii) the nature of the mutual shaping of people and the cultural contexts in which they live (Salter and Adams, 2016).

A ‘proverb’ is a fundamental part of language and culture (Raymond, 1956). It has been variously defined as an epigrammatic philosophical saying that conveys a lesson (Yankah, 2000); and as ‘a rich source of imagery and succinct expression; encapsulating abstract ideas and allusive wording usually in metaphorical form’ (Agbájé, 2002). Proverbs are generally regarded as repositories of folk wisdom (White, 1987). The study of proverbs (Paremiology) dates back to Aristotle (Dabaghi et al., 2010). Used worldwide, proverbs feature prominently in everyday discourse in many African settings. In particular, many African societies conserve and pass on beliefs, heritage, values, norms and other important information from generation to generation using proverbs (Brookman-Amissah, 1986; Lawal et al., 1997; Hussein, 2005). The societal impact of proverbs both in bygone and contemporary eras features prominently in the work of a plethora of Africanist scholars (Brookman-Amissah, 1986; Gyekye, 1995, 1996; Yankah, 2000; Odebunmi, 2008; Aguoru, 2012; Boyejo, 2011, Unpublished; Arinola, 2012), a review of which is beyond the scope of this paper. The proverb is a cultural artifact that has not been widely used in psychological research.

The connection between cultural artifacts and cultural norms has been noted by a growing body of research in Psychology [see reviews by Morling and Lamoreaux (2008) and Lamoreaux and Morling (2012) and research by An (2007), Tsai et al. (2007), and Salter and Adams (2016)]. The aforementioned and similar studies indicate that we can learn things about a culture by looking for patterns in its visual cultural products (e.g., newspaper and internet ads, children’s book illustrations, Black History Month displays). While the cited studies used comparative methods to tap cultural nuances, within-culture analysis can also yield psychologically useful information. Alimi (2012) noted how Chinua Achebe (An African proverbialist and renowned literary author) used proverbs as literary devices in his novels ‘Things Fall Apart’ and ‘Arrow of God’ to describe his characters’ appearances, actions, habits, inner feelings and thoughts. Proverbs have also been used to examine aspects of Chinese mental life (Zhong, 2008); the relationship between changes in song lyrics and cultural changes in psychological processes (DeWall et al., 2011); personality referrents in Romanian epitaphs (Curşeu and Pop-Curşeu, 2011), the psycho-cultural value of proverbial sayings in the work of Chinua Achebe (Aguoru, 2012).

Cultural affordances that make societies unique include factors such as motivations (Markus and Kitayama, 2010) and norms (Eid and Diener, 2001; Marcus and Sabuncu, 2016) that shape the elicitation (Vandello et al., 2008), communication (Crowe et al., 2012), lexica (Dzokoto and Okazaki, 2006), experience (Kitayama et al., 2006), psychological emphasis (Ryder et al., 2008), expression control (Matsumoto et al., 2008), and regulation (Ma et al., 2017) of emotion. It is therefore reasonable to assume that some of this cultural scripting of emotions will be reflected in a culture’s proverbs. Shi-xu (2009) advocates for researchers interested in understanding culture’s influence on emotions to strive to ‘identify the most pertinent, influential and enduring historical elements from cultural moral instructive discourse’ (p. 366). Proverbs fit that bill. Proverbs reflect the values of a society (Yankah, 2000). Research on emotion display rules and emotion regulation reviewed above has shown that values of a society drive emotion display rules (Matsumoto and Juang, 2013; Tsai et al., 2016). Thus, by extension, proverbs may be a cultural product from which to glean information about emotion display rules and emotion regulation practices.

A few studies have explored affect in proverbs. Kurt (1991) examined emotion referencing in cognitive metaphors underlying Judeo-Spanish and Turkish proverbs. Norrick (1994) identified various elements that were representations of expressed emotions in American proverbs, such as exaggeration, images, metaphors, the use of stern warning and impolite vocabulary. Lim (2010) found that emotions tended to be encoded in Malay proverbs as evidenced by various markers of affect. Interestingly, no study has as yet explored emotion in African contexts- contexts in which proverbs are so widely used that they are described as ‘the palm oil with which words are eaten’ (Achebe, 1958).

Exactly how useful are proverbs – age-old nuggets of folk wisdom some of which may have been coined in historic periods preceding modern inventions - in today’s Africa? Are they about as useful today as floppy disks? Hardly. African proverbs provide a window into dominant African worldviews, philosophies, and morality (Mbiti, 1969; Gyekye, 1987). More specifically, proverbs serve as mechanisms for conflict resolution (Kwakye-Nuako et al., 2017), reminders about the social hierarchy and social comportment expectations (Dzahene- Quarshie and Kambon, 2017). They constitute social commentaries on constructions of masculinity (Amfo and Diabah, 2017) and gender (Ahiakpor et al., 2017). Cultural change has occurred over the centuries as a result of influences such as western style education, globalization, rural-urban migration, and the introduction of christianity. Yet, many central values (e.g., kinship systems and ties, importance of spirituality, honoring elders, expectations in romantic relationships) persist (Gyekye, 1996; Goodwin et al., 2012; Darkwah, 2016). Motherhood, for instance, is still very highly valued even though Ghanaian demographic trends indicate societal changes in the context of childbearing. [Between 1998 and 2003 the median age of marriage in Ghana increased; reported age at first intercourse increased by 2 years; the fertility rate decreased from 6.4 to 4.4; Christians were more likely to have less than 3 children per family on average; and the age of first pregnancy increased (Agyei-Mensah, 2005; Heaton and Darkwah, 2011)]. As such, while specific elements may have changed, many underlying elements of what is considered to be good for an individual and Akan society have persisted over time. As such, messages contained in proverbs cannot be presumed obsolete.

Proverbs have an active presence in the contemporary Akan world. In everyday discourse, Akan proverbs serve as a socially acceptable means to be impolite (Anderson, 2017). In traditional settings, proverbs are still one of the verbal strategies used to communicate with chiefs as Yankah described in 1995. They are also featured in literary works of Ghanaian writers (e.g., Aidoo, 1993). Modern developments have expanded the ways in which proverbs are used and circulated. For instance, Akan proverbs are routinely used on Akan radio and television programs (Wiafe-Akenten et al., 2017); in Akan popular music (Agyekum, 2017); in Ghanaian choral music (Amuah and Wuaku, 2017); and in teaching in academic settings (Osseo-Asare, 2017). Regional proverbs have been featured as a task for contestants in a Ghanaian beauty pageant (Ghanaweb, 2011). For at least a decade, one Akan language radio station has run a weekly, hour-long program aired on Sunday evenings in which two competitors engage in a back-and-forth verbal duel of proverbs before a panel of judges. The rules for the program stipulate that appropriate proverbs must be given in response to the previous proverb by the opponent, no proverb can be repeated, and the proverbs have to exist - as in, no one is allowed to make up a proverb on the spur of the moment. Videos of elders engaging in similar friendly proverb mastery competitions have been posted on YouTube and circulate on WhatsApp. Essien (2015) interestingly advocated for a *decrease* in the use of proverbs and other literary devices in local language news radio programming because according to him, they distract listeners from the central points of the news stories. In sum, far from being a historical relic, proverbs permeate everyday life within and beyond Akan society.

### Objective of the Study

The objective of this study is to explore emotion in the Akan society of Ghana through the lens of proverbs as a cultural product. We posited that an examination of a compendium of Akan proverbs will point toward emotion norms and highlight salient approaches to emotion regulation in the culture. Adams (2005) briefly and indirectly hints at the connection between proverbs and culturally grounded behavior and attitudes in his investigation of enemyship and distrust/fear of social others in West African settings. He notes that belief in the existence of dangerous others in immediate social circles are mirrored by proverbs such as “if an insect bites you, it comes from inside your clothes.” Adams goes on to utilize a variety of quantitative data sources and a cultural comparative approach to demonstrate the difference in the cultural grounding of social relationships between West African and North American cultural settings.

Because of the limited amount of research in the area of proverbs and the African psyche, as well as cultural differences in the prevalence, utility, and functions of proverbs between various African and Western (the de facto control in comparative cultural psychology research) cultural contexts, we utilize a non-comparative, qualitative approach in our research. In contrast to a quantitative approach, we use thematic analysis to identify emotion rules promoted by proverbs. Qualitative approaches to investigative inquiry – such as thematic analysis- are particularly useful in illuminating meaning; understanding how and why aspects of specific contexts matter; and unpacking how things, systems, institutions, and cultures work (Patton, 2015). A recent APA task force recognized the significant contributions that researches employing qualitative methods of inquiry have made to the field of psychology (Levitt et al., 2018). The authors observed that the focus on single, often limited, data set yields rich and contextualized descriptions which are often bound to a context or setting. The non-comparative nature of the findings notwithstanding, qualitative findings have their own merits (including theory or social construct development; hypothesis testing; development of initial understandings of a phenomenon or illuminating current understandings of a social process).

The goal of the present study is not to compare the extent to which proverbs provide emotion discourse across different cultures. Rather, we seek to examine a single cultural context to generate a detailed description of complex ways in which this particular, understudied cultural setting may shape affect. In contrast to a comparative epistemology therefore, we use an exploratory, indigenous, cultural study approach to address our research question: the extent to which and the manner in which Akan proverbs address affect. Indigenous cultural studies operate from the assumption that because psychological processes and associated behavior can only be understood within their originating cultural systems, it is crucial to scrutinize the cultural settings that produce the psychological processes (Matsumoto and Juang, 2013). These processes include emotions, and for Akan culture, the cultural setting includes proverbs. As such, our study analyses the proverbs to understand how culture shapes emotions in the Akan.

Similar approaches are adopted in the humanities, especially sociolinguistics, literature, and African Studies. However, we deviate from cultural study and humanities approaches by focusing on psychological principles associated with these cultural influences. In particular, we draw from contemporary psychology theories about emotion and emotion regulation to guide our enquiry. We explore the cultural foundations which may be relevant for the grounding of emotional experience, expression, responding, and management within a specific cultural milieu.

The importance of our study lies in the research gap it starts to address. On the one hand, linguistic anthropology has examined emotion in language in African settings, but has not generated much psychologically useful information due to a predominant focus on morphology, syntax, and pragmatics. On the other hand, while the field of culture and emotion in general has made large strides in exploring the intersection between culture and emotion at a global level, Africa has been significantly under-represented. The goal of the present study is to provide a foundation upon which future research can be based.

### The Akan: People, Language and Values

The Akan constitute Ghana’s largest ethnolinguistic group and 47.5% of Ghana’s 24 million citizens (Ghana Statistical Service, 2012). Akans are mostly located in six of Ghana’s ten administrative regions (Agyekum, 2011). The Akan language is one of the most widely spoken and widely studied Ghanaian languages (Agyekum, 2011). It consists of 12 mutually intelligible dialects. Popularly spoken even among multilingual non-native speakers in Ghana, it is routinely used on local language radio stations in Ghana. The first Akan dictionary was developed by Christaller in 1933. The Bible has also been translated into some Akan dialects. Akan can also be found in Eastern and Central Côte D’Ivoire, Ghana’s francophone neighbor.

What does Akan culture value? Traditionally, Akan societies have been described as stratified; with age being one of the main determinant of social role and status. Older people are to be accorded honor and respect. Akan societies are also patriarchal, with gender being an important factor that influences status (Obeng and Stoeltje, 2002). National values data can be taken as a proxy for Akan values given many similarities in values across Ghana’s various ethnolinguistic groups. Ghana ranks higher on Power Distance, and lower on Individualism, Masculinity, and Long Term Orientation compared to the United States and Japan. Ghana’s mean Uncertainty Avoidance score is higher than the United States and lower than Japan’s (Hofstede Insights, 2018). In the World Values Survey’s Wave 5, Ghana ranked higher on traditional values, and lower on self-expression values, and lower on subjective well-being than many western countries (World Values Survey Database, 2018) While these top-down approaches are useful, bottom-up approaches provide complementary and contextual insight. Gyekye (1996) asserts that Akan culture values religion, communal values, marriage, children, traditional political authority, and wealth-building. Adjaye and Aborampah (2004) found that in Akan communities, extended families persist as important kinship structures despite the increase in single (nuclear) family homes due to urbanization and migratory trends, and that elders still play a pivotal role in the intergenerational transmission of cultural values. Extant literature on Akan values has not adequately explored what Akan culture values as far as emotions are concerned. We approach this research question via a bottom-up approach.

## Materials and Methods

### Emotion Referent Proverbs Identification and Selection

We analyzed seven thousand and fifteen (7,015) Akan proverbs compiled by Appiah et al. (2007) for emotion referents. This compendium of proverbs, gathered over several decades, is the largest and most comprehensive published work of Ghanaian proverbs to date (the previous largest source (Christaller, 1879) had only half as many). The proverb compendium *Bu Me Be, Proverbs of the Akans* has the original proverbs in Twi (an Akan language), followed by their English literal translations, and their manifest meanings (interpretations) in English. A bilingual (Twi and English) co-author read through each of the 7015 proverbs and created a database consisting of every:

(i) proverb or associated manifest meaning referencing affect (a specific emotion, or mood);

(ii) proverb or associated manifest meaning referencing affect expression (e.g., crying, smiling, weeping, laughing)

(iii) proverb or its manifest meaning alluding to the absence of affect (e.g., proverbs focusing on bravery and courage dealt with the absence of fear).

### Coding and Validation

Two hundred and eighty five proverbs had affect referents. Coding was conducted in multiple stages, each with two independent raters. Step 1 involved inductive coding to identify and categorize the specific emotions (such as sadness, fear, and love). Proverbs were also analyzed for cultural nuances (e.g., cultural descriptions; cultural prescriptions; and social context). Interrater agreement was 86.0%. Step 2 was deductive coding using Gross’s emotion regulation strategies as codes. Overall agreement between the coders was 71.9%. Step 3 comprised deductive coding using the six display rules identified by Matsumoto and Juang (2013) as a guide. Interrater agreement was 93.3%. For each of the coding steps, the task was to determine whether a particular code was present either in the proverb or in its meaning provided in English by the authors of the proverbs. Code generation at each stage of the analysis involved: (1) independent identification of codes by two coders; (2) code validation by the two coders; and (3) resolution of discrepancies through discussion. In Steps 2 and 3, the proverbs were also analyzed for affect management strategies and display rules that had not been identified by Gross and Matsumoto, respectively.

## Findings

We identified proverbs addressing positive and negative affect and associated behaviors. Negative affect addressed in the Akan proverbs were coded into the following categories: *Hurt/Pain/Suffering; Fear; Hatred/Resentment/Contempt; Sorrow/Grief; Anger; Shame/Disgrace; Regret/Disappointment; Dislike; Jealousy/Envy; Worry/Anxiety; Pride*^1^, *and Distress*. Some of these negative emotions were simultaneously seen as negative and precursors to other negative emotions in proverbs such as “*greed brings suffering.*” Positive emotions and associated behaviors included *Love; Pleasure/Happiness; Bravery/Courage* (coded as positive emotions due to their focus on the absence of fear)*; Sympathy/Compassion; Laughter;* and *Gratitude.* The ratio of negative to positive emotion-focused proverbs was 4.8:1. Category examples are summarized in Tables 1a,b.

**Table 1a:** Examples of negative emotion-focused proverbs.

Code	Percentage (*N* = 285)	Proverb	Translation in English	Meaning
Anger	7.7	#1037: Abofuo de asεmtenten na εnam	Anger brings with it a long tale.	Anger makes people talk too much.
Dislike	1.8	#4521: Nnipa kyiri aponkyerεne nso wɔnom ne ho nsuo	People dislike the frog but drink the water it is in.	You may have to spend time with someone you dislike
Distress	0.7	#3040: Kasabodin nti na kɔkɔsakyi yam tuiε	Owing to constant slander the vulture head constant diarrhea.	Constant slander causes distress
Fear	11.6	#1447: Wode suro di adona a, yede wo sekan dwa apetebie	If you fear to show displeasure they use your knife to skin a striped squirrel	If you are not tough, people will take advantage of you in small things
Hatred/Resentment/Contempt	8.4	#6050: Ɔtanhunu yε ya	Hatred without cause is bitter	It is hard to be hated when you have given no cause for it.
Hurt/Pain/Suffering	24.2	#2426: Agohia sene hia pa	Having no one to play with is worse than poverty.	Loneliness is one of the worst forms of suffering
Jealousy/Envy	1.1	#1972: Ɛdɔm ani sa odedeni	The masses are always critical of a popular person.	Envy never ends
Pride	3.2	#4323: W’ani tra W’anintɔn a, woyera	If you look beyond your eyebrows you get lost.	Pride comes before a fall
Regret/disappointment	2.8	#2782: “Mehunuiε a anka”, na aseε awie	“Had I known,” is always too late.	Vain regrets
Shame/disgrace	6.7	#2313: Fεreε ne wuo, fanyinam wuo	Shame and death, death is preferable	Shame and death, death is preferable
Sorrow/Weeping/Crying/grief	7.7	4486: Onipa nsu kwa	Man does not weep for no reason	Man does not weep for no reason
Worry/Anxiety	1.8	#6695: Yεbεwu nti yεnna?	We will die, but does that mean we should not sleep?	There is no point in worrying about the inevitable


**Table 1b:** Examples of positive emotion-focused proverbs.

Code	Percentage (*N* = 285)	Proverb	Translation in English	Meaning
Bravery/Courage	2.8	#3265: Wo koko yε duru a, wonnwura ogya mu	Even if you are brave, you can’t enter a fire.	There are limits even to bravery
Gratitude	2.1	#5340: Se kɔtere bɔ tɔfa ma wo a, nka sε εsua εfiri sε deε ne nsa tumo bɔ ara ne no	If a lizard squeezes mashed yam to give you, don’t say that there is not enough, because that is all its hand can press.	Be grateful when people do the best they can for you and don’t expect more than they have to give
Laughter	1.1	#687: Obi nsere hia	No one laughs at want	No one laughs at want
Love	4.6	#1940: Ɔdɔ mu nni nkekaawa	Love is pure	Love is pure
Pleasure/Happiness	3.5	#4127: Ɔman mu yε dε a, ene wo fie	If there is something good in the state, then it is (because) your house (is in good order).	Happiness begins at home
Sympathy/Compassion	1.4	#3642: Ɔkra se: awerεkyekyerε nti na ɔde ne ho twitwiri nnipa ho	The cat says: it is for consolation that it rubs itself against man.	You make friends because you need sympathy


The proverbs utilize a variety of formats including declarations (*Loneliness is one of the worst forms of suffering)*; predictions [e.g., *If a male cricket gives a fatal blow, some of it gets in his eyes* (A warning against fighting or litigation: You will suffer even if you win)]; references to folktales [e.g., *Because of shame, Ananse (Father Spider) wears an antelope skin hat to beg for help in weeding his farm* (Said of someone who tries to hide what he is like because he wants to get something out of you)]; descriptions (e.g., *Anger is like a stranger, it does not stay in only one person’s house*); and comparisons [e.g., *There are many kinds of weeping: weeping from a game is one thing, weeping from beating is another, weeping from death is yet another, and pretended weeping another* (There is a right time for every action)].

Thematically, the proverbs endorse specific regulation strategies, display rules, emotion-specific directives and nuances (cultural prescriptions, cultural descriptions, positive and negative consequences of specific emotions) and the Akan social context (such as emotion norms, social hierarchy, and social interactions). These are discussed below, with our proverb examples provided in italicized English and their manifest meaning in parentheses (except in cases where the literal and manifest meanings are the same).

### Emotion Regulation

Emotion regulation strategies featured prominently in our data (see Table 2). Proverbs such as “*We will die, but does that mean we should not sleep?*” (There is no point in worrying about the inevitable) reference change in cognition. Some proverbs advocated for *response modulation* [e.g., “*A great man weeps through his pipe*” (A great man suffers in silence)]. Proverbs such as “*A person full of sores does not go into fellowship with anyone*” (If you know you are vulnerable, you avoid those who may hurt you) reference *situation selection.* “*If you are unhappy, you say; I am going to Dawu to drink*” (If you are unhappy, you look for happiness outside your own community) reference *Situation modification.* Also relevant to emotion regulation, some proverbs focus on negative consequences of emotion [e.g., “*If one person alone eats the honey, it awakens his stomach (he gets diarrhea)*” (Greed brings suffering)]; while others focus on positive consequences [“*Remember your creator in the days of your youth, before the bad times come*” (Always be grateful to your benefactor and he will continue to help you)].

**Table 2:** Examples of proverbs for emotion regulation.

Codes	Percentage (*N* = 285)	Proverb	Translation in English	Meaning
Change cognition	11.9	2617: Ehia wo a, nwu	If you are in need, don’t die.	Don’t despair in adversity
Normalization^∗^	3.2	5487: Asεm a εwo anisoɔ na yεde ko daeε mu	If we are anxious we have bad dreams.	We worry about what concerns us most
Response modulation	22.5	2314: Fεreε nti na Agya Ananse de ɔtwe kyε hyε srε adɔ	Because of shame Father Spider wears an antelope skin hat to beg for help in weeding his farm.	Said of someone who tries to hide what he is like because he wants to get something out of you
Situation modification	0.4	4328: W’ani nnye a, na wose: “Merekɔ Dawu asanom”	If you are unhappy, you say: “I am going to Dawu to drink.”	If you are unhappy, you look for happiness outside your own community
Situation selection	3.9	592: Obi ni ayie ase na obi su ne ni	At someone else’s mother’s funeral you weep for your own mother.	Someone else’s sorrow reminds you of your own


*^∗^Normalization is a form of change in cognition.*

Proverbs contained messages that normalized particular emotion experiences. Such proverbs refer to cultural narratives about life including the normality of suffering [*The deserted town’s small stones say: “it is because of death that we have been scattered*” (People suffer from circumstances beyond their control)]; the naturalness of experiencing anxiety in particular contexts [e.g., *If we are anxious we have bad dreams.* (We worry about what concerns us most)]; the typicality of emotions and/or consequences associated with specific social interactions [*If you feel for the young duiker, you eat your soup without meat* (If you are too sympathetic, you suffer for it)]; and the futility of agonizing [*We will die, but does that mean we should not sleep?* (There is no point in worrying about the inevitable)]. Such normalizations have the potential to allow the listener to reappraise emotion-eliciting situations. Proverbs thus furnish a cultural script for coping with negative situations by providing connections between individual situations and experiences of non-specific others within the same cultural context. In a few cases, the proverbs come across as rather brusque [e.g., *In normal times, they say that the forest devil is a witch, then how much more so when he is found sitting on the odum tree that bears the fruit of dwarfs?* (If you fear something in normal times, how much more so in times of trouble)], potentially serving the additional function as a culturally sanctioned tool that can be used to be impolite (see Anderson, 2017).

### Emotion Display Rules

While no proverbs touch upon specific emotion display markers, some proverbs address preferred action tendencies with respect to particular emotions and social contexts (see Table 3). Shame, a proverb declares “*is not a Northern smock that we sew and wear*” (We do not advertise our shame). Should an elder need to cry, another proverb dictates that “*he cries in his head*” (A senior man does not give way to grief in public). We surmise that the cultural expectation that particular emotions should be expressed privately is linked to the loss of status and respect in the eyes of others. This makes sense given that Ghana scores relatively high on Power Distance. In hierarchical contexts, the loss of status is particularly salient, since it disrupts the social order.

**Table 3:** Examples of proverbs related to display rules.

Codes	Percentage (*N* = 285)	Proverb	Translation in English	Meaning
Amplification	7.4	1447: Wode suro di adona a, yede wo sekan dwa apetebie	If you fear to show displeasure they use your knife to skin a striped squirrel	If you are not tough, people will take advantage of you in small things
Deamplification	20.7	6695: Yεbεwu nti yεnna?	We will die, but does that mean we should not sleep?	There is no point in worrying about the inevitable
Masking	5.6	1041: Abofuo nti na ɔhemmaa fua papa	Because of anger the Queen Mother holds the fan	The Queen Mother holds a fan and waves it to show she is annoyed. We use the symbol to illustrate the reality.
Neutralization	0.7	5487: Asεm a εwɔ anisoɔ na yεde kɔ daeε mu	A problem that is on the mind is what we dream of.	If we are anxious we have bad dreams. We worry about what concerns us most


Proverbs communicating specific emotion display rules were complemented by others that provide an explicit commentary on undesirable emotions: pride and shame. Proverbs featuring pride communicate that pride – at the level of the individual- is a sign of arrogance and comes before a fall in a variety of ways including :“*It is only pride which causes a lizard not to move when you shoot an arrow in it*” (Pride may lead to bravado); “*Know-all knows nothing*”(If you pride yourself on your wisdom, it is a sign of ignorance); and “*There is nothing more in pride than getting involved in trouble*” (Pride comes before a fall). While many western cultures consider pride to be a positive emotion, this is clearly not the case for the Akan culture. Proverbs such as “*Congratulations for giving birth can never end*” (People are always pleased at success) do endorse the acknowledgment success. However, the dominant proverb narrative is one in which success should be celebrated, but not evolve into pride. Another emotion noted as undesirable was shame, a message communicated via proverbs including: “*Shame and death, death is preferable”; “If a paramount chief who is above destoolment becomes ashamed, he dies through his mouth, i.e., he consumes poison* (A great man prefers death to shame); and “*Secret criticism hurts more than a wound*” (To be hurt physically is less painful than being shamed). Such proverbs communicate expectations that Akans avoid situations that would generate shame, and not display pride.

### Akan Emotion Values and Beliefs

The symbolism in the analyzed proverbs includes references to traditional values and practices such as religion, death as a means of contextualization, and the valuing of the group above the individual. Below, we discuss values and beliefs about emotions highlighted by our proverb set that are relevant to emotion regulation and display rules.

#### Emotion Limits

The collective proverb emotion narrative includes distinctions in limits of emotion. On the one hand, proverbs communicate naturally occurring limits [e.g., “*He who weeps cannot weep beyond the cemetery*” (Even sorrow has its limits); and “*Even when you are brave, you can’t enter a fire*” (There are limits even to bravery)], suggesting that some affect types eventually run their course. On the other hand, proverbs – such as “*If a man suspects that his wife commits adultery, he should go and suspect the fallen tree across the path to his wife’s farm*” (However jealous you are there must be limits to your jealousy) and “*Too much benevolence brings suffering to the generous*” communicate that some emotions and associated actions should have limits.

#### Prescriptions (Mandates)

“*If God is going to satisfy you, it does not matter if you take a big portion of fufu*^2^ (Be content with what you have).” “*Remember the past” we say to those who are ungrateful*. (You should always show gratitude for past favors). “*Salt should not praise itself saying* “*I am sweet*”” (It is not good to be arrogant nor to praise yourself to others). Proverbs such as these are instructional in nature, communicating expectations about specific affect-related behaviors, see Table 4.

**Table 4:** Sample of proverbs related culturally specific factors.

Codes	Percentage^∗^ (*N* = 285)	Proverb	Translation in English	Meaning
Cultural description	33.3	2023: Adu Takyi Bɔtonkuruwa: ɔkoto didi a, na εyε aponkyerεne ya	The hero Adu Takyi Bɔtonkuruwa: says: “If the crab eats, the frog is jealous”	Many people are jealous of the success of others
Cultural prescription	9.8	4920: Ɔpanin su a, ɔsu ne tirim	If an elder cries, he cries in his head.	A senior man does not give way to grief in public
Negative consequence	32.3	1042: Abofuo yε ɔbrammiri	Anger turns a man into a strong man	Anger makes a weak man violent
Positive consequence	6.0	1937: Ɔdɔ de bɔne kyε	Love forgives shortcomings	Love forgives shortcomings
Social context	28.8	4330: “Ɔni me da bi a, ɔnni me bio”, yε ya	“He knew me once, but he does not know me anymore” is bitter	The loss of an old friendship is bitter
Social hierarchy	8.4	4899: Ɔpanin fεre ne ban a ɔnsuro no	An elder respects his child but does not fear him	A senior man is not afraid to tell anyone junior to him the truth as he sees it


*^∗^The total in the percentage column do not add up to 100%. Some proverbs were coded under more than one code.*

#### Emotion Descriptions

A subset of proverbs were descriptive in nature, using linguistic tools such as metaphors and similes to communicate philosophies of what emotions are like (e.g., *Anger is like a stranger, it does not stay in only one person’s house*), and what people experiencing that emotion do [e.g., *If a person loves you, (s)he loves you with all your nonsense* (You don’t judge those you love, but love everything about them)].

### The Akan Socio-Emotional Context

In this section, we address themes observed in the proverbs that relate to the social context. While a few proverbs referenced gender and age (child, adult) these were much less prominent than the themes discussed below.

#### Socioeconomic Class

A group of proverbs address the intersection between socioeconomic class and affect. These proverbs suggest that socioeconomic class afforded the wealthy particular affect experiences distinguishable from those who were less well off. These included “*A poor man’s gunfire passes over a rich man’s ears*” (A rich man does not fear a poor man); “*Even the ocean that is deep has an excess of salt, how much more the shallow ditch*” (If a great man suffers, how much more the poor man); and “*If you are in need, one hand lies on the belly of another*”^3^ (The poor cannot afford pride). While the specific connections differ, the local narrative of an affect- socioeconomic class relationship mirrors findings from psychological science. Previous emotion research has demonstrated a link between emotion dysregulation and poverty (Kliewer et al., 2017). Subjective well-being has been linked to income (Diener et al., 2010).

#### Emotions in Private and Public Spheres

Proverbs address individual-level affect [e.g., “*If you are hungry, it is only you who feel it*” (You alone have to bear your own suffering)]; individual responsibility for some antecedents [e.g., “*If a person is unhappy, it is his own fault*” (We are often the cause of our own discontent)], and individual differences [e.g., “*If any great animal dies, the vulture puts on white (rejoices)*” (One man’s sorrow is another man’s joy)]. The body of proverbs also includes directives to learn from the emotion-related experiences of others [e.g., “*Obiri, look at what is happening to Oben*” (Take warning from the sufferings of your friends)]; directives to manage the emotions of others [e.g., “*We mix hot water with cold water*” (If someone is angry you try and cool them down, and do not make it worse)]; and reminders that individual actions can impact others negatively [e.g., “*If you have a dog as a member of your family, you are never without tears*” (A troublesome relation causes endless worry), and “*You should always keep your promises to children or they will be very upset*”)]. Impact on others can be deliberate, or accidental as highlighted in “*A funeral announcer causes them to kill a witch*” (One action unwittingly leads to another person’s suffering). Collectively, these types of proverbs address the intersection between the individual and the social. While emotions are certainly relevant to the individual, they are also relevant to the group that the individual is a member of. As such, the cultural narrative captured by the analyzed proverbs includes responsibility for one’s own emotions, and in some contexts, those of others.

#### Social Hierarchy

Expectations about maintaining decorum – especially for those of high status – were captured in proverbs focusing on behaviors such as public grief. Proverbs (including some addressed in categories above), mention high status position such as chief, paramount chief, and elder. Others mention servant/slave/employee status [e.g., “*A slave taboos the best palm nuts*^4^” (A slave fears to take the best of anything); and “*If you keep company with a leopard, you no longer fear anything in the bush*” (If you are friends with a powerful man, you don’t need to fear anyone)].

#### Emotional Contagion

The potency of an individual’s emotions to influence the social world is captured by proverbs highlighting emotional contagion. Proverbs such as “*Weeping is contagious*” and “*Anger is like a stranger, it does not stay in only one person’s house*” serve as a reminder that affect can be spread through the community via exposure to someone else’s emotion. One proverb in this category makes the distinction between this quality and other aspects of life that do not transmit across the community in a similar manner thus: “*If your mother dies, you don’t die, but if she is shamed, you are also shamed*” (Shame is worse than death).

#### Friendship and Enemyship

In addition to references to various kin, the proverbs highlight other interpersonal relationships relevant to Akan social functioning. Proverbs such as “*He knew me once, but he does not know me anymore is bitter*” (The loss of an old friendship is bitter); and “*The cat says it is for consolation that it rubs itself against man*” (“You make friends because you need sympathy”) focus on emotion in the context of friendship. In contrast, proverbs such as “*If those who hate you happen to know the inside of your case, if you talk salt, it turns to pepper*” (A person who hates you will always do their best to misinterpret you) and “*An army fears an army*” (You fear your enemies) focus on emotion in the context of enemyship. Taken together, these proverbs portray a social world that consists of supportive and potentially dangerous personal relationships (see Adams, 2005 for a review of enemyship in Ghana).

#### Social Harmony

Social harmony is relevant to many of the proverbs that addressed the social context. In addition, some proverbs addressed it explicitly e.g., “*The brother of want is peacefulness*”; “*If a male cricket gives a fatal blow, some of it gets in his eyes*” (A warning against fighting or litigation: You will suffer even if you win), and “*Anger drives something good from the house.*”

## Discussion

Altogether, emotion-focused proverbs (such as those showcased in our results) construct a philosophy of expectations – albeit incomplete- about how Akans ought to experience and express feeling. In some cases, proverbs articulate expectations about display rules – especially whether emotions should be hidden or expressed in public. In others, they provide cognitive frames relevant to the construction of meaning (e.g., what is normal in the experience of specific emotions; comparisons of emotion to death) from emotion-eliciting events in a variety of social interactions and life experiences (e.g., suffering) which can facilitate cognitive reframing and reappraisal. Proverbs address positive and negative consequences of emotion. They also promote particular, response-focused patterns of emotion regulation (situation selection, response modulation, and situation modification).

Often using symbolism that draws from physical objects and social occurrences in everyday life, the Akan narrative about emotions is woven around culturally salient elements such as interpersonal interactions. Certainly, emotion management is central to many cultural groups including the Akan people, for whom interpersonal associative ties of kinship and other affiliative bodies are central to constructions of identity, and associated behavior and decision-making. While psychological theory makes distinctions between emotion, emotion episodes, and mood, such distinctions are not evident in Akan proverbs. Akan proverbs seem to support an ‘anger-in’ (see Novin et al., 2012) approach, and discourage an ‘anger-out’ strategy. They also promote expression of positive emotions by highlighting personal (e.g., *a cheerful person does not arouse resentment*), familial (*love brings a good manner of living to the home*) and societal (*what is enjoyable is enjoyed by all*) benefits. Relevant to the preservation of social harmony, this trend is consistent with previous research that notes promotion of socially engaging emotion as a feature of interdependent cultural contexts, and a promotion of socially disengaging emotions of a feature of independent contexts (Kitayama et al., 2006). We posit that the discouragement of public expressions of grief and anger are relevant to the maintenance of social hierarchy since they decrease the likelihood of loss of face in high status individuals.

Including a bottom-up approach in our data analysis unearthed emotion-related nuances specific to the Akan proverbial context. Proverbs address dangers of emotional contagion of undesirable emotions such as shame; the expectation that particular emotions have or should have limits; and the intersections between socioeconomic class, social status, and various types of interpersonal relationships with affect. Contrary to our expectations, gender and developmental stages were not prominent in our data. Collectively, the proverbs reflected construction of a social world that involves kinship, friends, and enemies, and an emotion world that involves action, social interaction, and personal effort (in terms of adjusting one’s emotion displays and actions to social expectations). They communicate a concept of emotion that is not simply felt, but performed.

It is striking – but not surprising – that only 4% of Akan proverbs address emotion. The relatively minimal focus on emotion in the larger proverb collection mirrors the minimal explicit focus on emotion in Akan daily life vis-a-vis other concerns. In the United States people are socialized to talk about their feelings, think about their subjective well-being, and identify and seek what makes them happy. Such practices do not occur to the same extent in Akan and other Ghanaian societies. Ghanaian college students scored significantly lower than United States college students on a self-report measure of Emotional Attention, rating emotions as less important to attend to (Dzokoto, 2010). Compared to a college student sample from the United States, Ghanaian college students used fewer emotion words in written descriptions of emotionally significant events (Dzokoto et al., 2013). While emotions are not completely unimportant for the Akan, they are not verbally elaborated or valued in the same manner as the United States. Their relative paucity in the proverb collection is a reflection that other aspects of Akan values and practices are elaborated in greater detail.

### Limitations, Conclusion, and Future Research

Understanding cultural narratives about emotion helps document cultural philosophies. These cultural standards of expectation can inform emotion behaviors and emotion regulation strategies which influence behavior and cognition. Also, the fit of individual emotion beliefs and practices with the cultural milieu has implications for physical and mental well-being (see for example Potthoff et al., 2016). Our study demonstrates that Akan proverbs, which serve as a repository of culturally important values and norms, include maxims that favor particular emotional norms and regulatory practices consistent with the preservation of social harmony, prevention of loss of face, and establishment of often value-based standards for the experience and expression of emotion in everyday life. Akan proverbs thus form part of a cultural narrative that dictates how Akans ought to navigate and manage their emotions. A ubiquitous tool in a historically oral culture, proverbs have so far been under-utilized as a research resource in Cultural Psychology.

What our study fails to do, which should be explored in future research, is the actual impact of proverb messages; in other words, the extent to which, and processes through which proverbs’ emotion messages are socialized, and whether they are in fact internalized and demonstrated. For instance, it is unclear whether real time experiences, attitudes, beliefs, and values mirror those addressed in proverbs, or whether Akans perpetually disregard proverb messages, hence needing to be constantly reminded about a culturally utopian state of affairs. Our study also does not quantitatively examine the structure of emotion meaning components [such as appraisals and action tendencies, which can be assessed with tools such as the GRID questionnaire (Fontaine et al., 2013)]. Future research should explore these questions in Akan and other African groups, since – per an Akan proverb- “*A matter which troubles the Akan people, the people of Gonja take to play the brεkεtε drum.*”

## Author Contributions

VAD designed the study and wrote sections of the paper. AO-T was involved in data coding and writing. JK extracted the data and was involved in coding and data interpretation. MT-A revised and edited the manuscript. DAA and DA were involved in data extraction checks and initial drafting of sections of the literature review.

## Conflict of Interest Statement

The authors declare that the research was conducted in the absence of any commercial or financial relationships that could be construed as a potential conflict of interest.

## Footnotes

1 See emotion Display Rules section.

2 A kind of food.

3 Refers to the cultural hand gesture for pleading.

4 Used for making local alcohol (palm wine).
